# High-Performance Complementary Electrochromic Device Based on Iridium Oxide as a Counter Electrode

**DOI:** 10.3390/ma14071591

**Published:** 2021-03-24

**Authors:** Tien-Fu Ko, Po-Wen Chen, Kuan-Ming Li, Hong-Tsu Young, Chen-Te Chang, Sheng-Chuan Hsu

**Affiliations:** 1 Department of Mechanical Engineering, National Taiwan University, Taipei City 10617, Taiwan; chahlesko@iner.gov.tw (T.-F.K.); hyoung@ntu.edu.tw (H.-T.Y.); 2 Division of Physics, Institute of Nuclear Energy Research, Taoyuan City 32546, Taiwan; ctechang@iner.gov.tw (C.-T.C.); g923319@iner.gov.tw (S.-C.H.)

**Keywords:** iridium oxide (IrO_2_) film, nickel oxide (NiO) film, electrochromic device (ECD), cathodic arc plasma (CAP)

## Abstract

In complementary electrochromic devices (ECDs), nickel oxide (NiO) is generally used as a counter electrode material for enhancing the coloration efficiency. However, an NiO film as a counter electrode in ECDs is susceptible to degradation upon prolonged electrochemical cycling, which leads to an insufficient device lifetime. In this study, a type of counter electrode iridium oxide (IrO_2_) layer was fabricated using vacuum cathodic arc plasma (CAP). We focused on the comparison of IrO_2_ and NiO deposited on a 5 × 5 cm^2^ indium tin oxide (ITO) glass substrate with various Ar/O_2_ gas-flow ratios (1/2, 1/2.5, and 1/3) in series. The optical performance of IrO_2_-ECD (glass/ITO/WO_3_/liquid electrolyte/IrO_2_/ITO/glass) was determined by optical transmittance modulation; ∆T = 50% (from T_bleaching_ (75%) to T_coloring_ (25%)) at 633 nm was higher than that of NiO-ECD (ITO/NiO/liquid electrolyte/WO_3_/ITO) (∆T = 32%). Apart from this, the ECD device demonstrated a fast coloring time of 4.8 s, a bleaching time of 1.5 s, and good cycling durability, which remained at 50% transmittance modulation even after 1000 cycles. The fast time was associated with the IrO_2_ electrode and provided higher diffusion coefficients and a filamentary shape as an interface that facilitated the transfer of the Li ions into/out of the interface electrodes and the electrolyte. In our result of IrO_2_-ECD analyses, the higher optical transmittance modulation was useful for promoting electrochromic application to a cycle durability test as an alternative to NiO-ECD.

## 1. Introduction

Electrochromic devices (ECDs) have attracted considerable attention because they have tremendously promising applications in energy-saving smart windows that can enhance the optical properties and durability reversibly upon the application of a Direct Current (DC) voltage [1,2]. Electrochromic (EC) materials applied on smart windows can easily dominate the indoor illumination and effectively decrease the air-condition loading of buildings [3,4]. Furthermore, ECDs can save renewable energy and cause a persistent reversible color change upon the application of a small voltage [3,4] to reduce the energy consumption significantly; therefore, they are an extraordinary material providing some unique advantages such as larger optical modulation and better cyclic stability against sunlight exposure, for smart windows in a green building environment [5,6]. In general, ECD consists of a five-layer structure such as TCO/EC/IC/CE/TCO layers, where TCO, IC, and CE are transparent conducting oxide, ion conducting layer, and counter electrode, respectively [7,8,9]. In recent years, electrochromic materials have attracted considerable research interest in numerous metal-oxides, including molybdenum trioxide (MoO_3_), vanadium oxide (V_2_O_5_), niobium oxide (Nb_2_O_5_), and titanium dioxide (TiO_2_) [9,10]. In addition to the oxides, conducting polymers are also widely studied in electrochromic devices [11,12]. However, typically, complementary electrochromic devices include anodic and cathodic electrodes in a multi-layer. Tungsten oxide (WO_3_) film is the most commonly used electrode material and is complementary to an anodic layer of NiO or another extraordinary material such as IrO_2_ [13,14,15]. Electrochromic IrO_2_ and NiO films have been manufactured by diverse procedures such as sputtering [16,17], pulsed laser system [18], cathodic electrodeposition [19], chemical vapor deposition [20,21], thermal evaporation [22,23,24], and sol-gel [25,26,27].

Here, a type of counter electrode iridium oxide (IrO_2_) layer was fabricated using vacuum cathodic arc plasma (CAP). We compared two anodic coloration material of IrO_2_ and NiO films [13,14] which showed IrO_2_ films were better to enhance electrochromic properties than NiO. Cathodic arc plasm (CAP) fabricated procedure has been widely used in several types of films owing to the outstanding features of the arc plasma fabricated from cathode spots. In this method, the macro-particles (MPs) are released on account of the severe plasma-liquid pool influence on the cathode spots, and MPs sticking to the films make worse the properties of thin films. The infamous macro particle situation is the key reason why CAP system is not commonly applied in high-tech field. We implemented two ways to improve the quality of materials. One is to reduce macro-particles when arc discharge from a random (insufficient and outer magnetic field) to an arc (at axial magnetic field) showed that immediately improve the quality of nitride coating [28]. The result showed that less macro particles are thrown out using high horizontal magnetic field and increasing clear spot velocity [29]; another is by means of Theorton deposition [30], the composition of loose-packing structure at high working pressure condition. Arc plasms can be controlled in high pressure, and the structure may appear self-organized and lessen macro particle size [31]. Recently, researchers have investigated more study in monolithic coatings than higher-performing multilayers. The formation of electrode structure is controlled by the flow of argon (for insertion) and oxygen (reaction) [8]. In the recent years, P. W. Chen et al. [32] investigated in vacuum cathodic arc plasma (CAP) to fabricate all solid-state electrochromic devices (ECDs) with tantalum oxide (Ta_2_O_5_) as ion conductor layer. It emphasized on manufacturing Ta_2_O_5_ film by CAP with various gas ratios of oxygen and argon. K. Li et al. [33] suggested that indium–zinc–tin oxide (IZTO) films make use of the controlling powers of DC magnetron sputtering to improve the surface properties of ITO as a transparent electrode. We have manufactured ECD composing of a WO_3_ electrode film on IZTO/ITO/glass and a counter-electrode (Pt mesh) using 0.2 M LiClO_4_/PC solution. P. W. Chen et al. [8] used CAP deposition to make the porous surface structure of WO_3_/NiO films to upgrade the electrochromic performance. They found that the thickness of WO_3_ layers is an essential factor of ECDs for optical and electrochemical properties. However, the NiO film used as a counter electrode in ECDs is more susceptible to degrade upon prolonged electrochemical cycling which leads to insufficient device lifetime than IrO_2_ film.

In this study, we used a CAP technology to deposit IrO_2_ films provided porous surface structure to elevate electrochromic properties and promote switching speeds. The aim of this work was to compare between IrO_2_ and NiO films; however, a systematic study of the effect of the Ar/O_2_ mixing ratio by means of CAP is lacking. This fabricated technology is exclusive for high deposition rates with a low-cost method and can be used to fabricate diverse transition metal-oxides having nanostructures with a morphological phenomenon. We focused on the influence of various Ar/O_2_ gas-flow ratios with an IrO_2_ electrode as compared to NiO on the diffusion behavior of ion insertion/extraction, material structure, surface morphology, transmittance optical modulation, and durability test.

## 2. Materials and Methods

### 2.1. Preparation of Transparent and Electrochromic Electrodes and Electrolyte Materials

**Figure 1:**
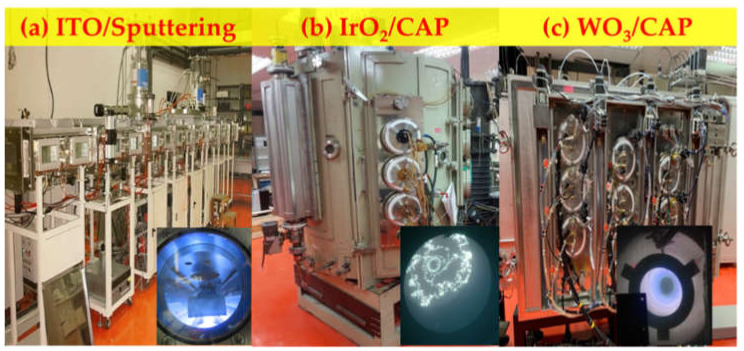
Complementary electrochromic device deposited system (**a**) indium tin oxide (ITO), (**b**) IrO_2_ electrode, and (**c**) WO_3_ electrode.

In this study, we used of method of CAP deposition technology as an alternative to sputtering in order to achieve high deposition rates at a low cost of producing EC films based on NiO and IrO_2_ electrodes for ECD applications. We prepared a series of IrO_2_ (Sample 1–3) and NiO films (Sample 4–6) under increasing Ar/O_2_ gas-flow ratios (1/2, 1/2.5, and 1/3) as anodic layers, which were deposited on a 5 × 5 cm^2^ indium tin oxide (ITO) glass substrate with a resistance of 6 Ω/cm^2^. The deposition process is presented in Table 1. Prior to the deposition of each electrochromic layer, the ITO-coated glasses were cleaned ultrasonically with ethyl alcohol and deionized water for 15 min to remove contaminants. Both the IrO_2_ and NiO electrodes were implemented via cathodic arc plasma (CAP) by using a pure metallic iridium (Ir) target (99.95%) and nickel (Ni) target (99.95%) with a diameter of 76 mm, respectively, in the vacuum chamber. Moreover, WO_3_ as the cathodic layer using a tungsten (W) target (99.95%) was fabricated, as listed in Table 2 by using the CAP technology. For the electrodes to ensure the lithium (Li^+^) ion transport, we were carried out a liquid electrolyte system containing lithium perchlorate (LiClO_4_) and propylene carbonate (PC, C_4_H_6_O_3_) at a weight ratio of 0.1325 [4]. The complementary electrochromic devices (ECDs) consisted of five superimposed layers with an ITO (300 nm)/IrO_2_ (100 nm) /LiClO_4_-PC (100 um)/WO_3_ (100 nm)/ITO (300 nm) structure and fabricated by deposited system respectively in Figure 1.

### 2.2. Measurements and Characterizations

The electrochemical properties of the electrochromic electrodes were measured using cycle voltammetry (CV) (model PGSTAT30, Autolab, Utrecht, The Netherlands) in a three-compartment system containing the abovementioned electrodes as the working electrodes (IrO_2_/ITO/glass and NiO/ITO/glass) and Ag/AgCl as the reference electrodes, and Pt foil as the counter electrodes. Sample 1–6 carried out electrochemical cycles in the 0.5-M LiClO_4_-PC solution at −0.5 V to 2.0 V and a potential sweep rate of 100 mV/s. The optical transmittance spectra of the colored and the bleached states were obtained using an ultraviolet-visible (UV-Vis) spectrophotometer (model DH-2000-BAL, Ocean Optics, Dunedin, FL, USA) in a wavelength range from 300 nm to 1000 nm. The crystalline structure was characterized by a high-resolution X-ray diffractometer (HRXRD, Model D8, Bruker AXS, Billerica, MA, USA) equipped with a CuKα (λ = 0.154 nm) radiation source over a 2θ scan region of 20° to 70°. It is provided with the essential accuracy and precision to measure the broadening and the relative peak. The surface morphological properties were examined with a field emission scanning electron microscope (FE-SEM) (Model S4800, Hitachi, Tokyo, Japan) operated at 15 kV. 

## 3. Results and Discussion

### 3.1. IrO_2_/ITO and NiO/ITO Films: Ionic Diffusion

**Figure 2:**
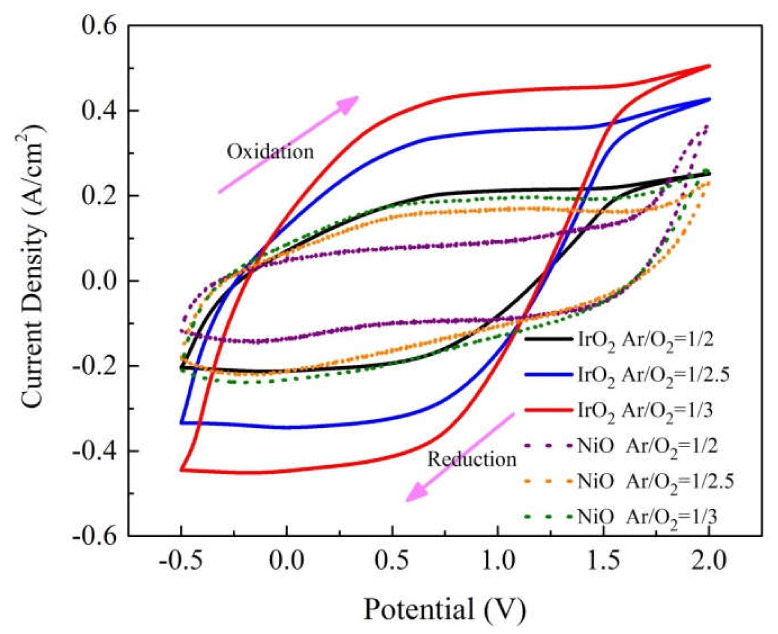
Comparison of 25th cycle CV curve of IrO_2_ (solid line) and NiO (dotted line) electrode films at a potential sweep rate of 100 mV/s.

**Figure 3:**
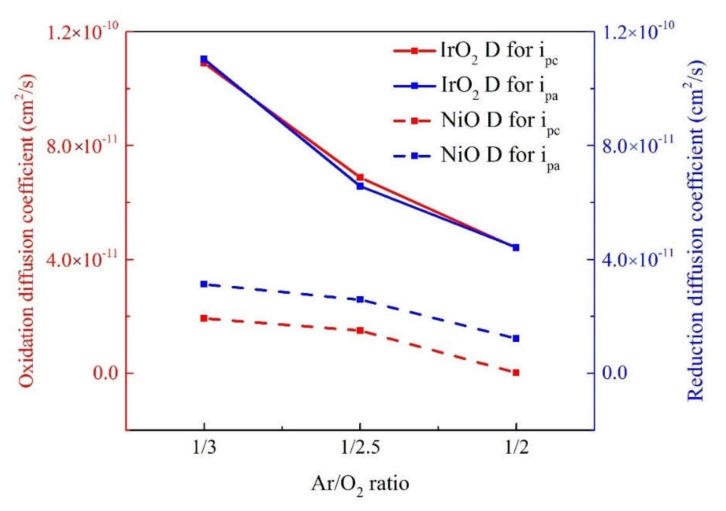
The comparison of diffusion coefficients of IrO_2_ and NiO electrodes with various Ar/O_2_ mixing ratio.

In this work, we utilized the cyclic voltammetry (CV) method and the Randles–Servick equation to calculate the ionic diffusion coefficients [34,35]:

J_p_ = 2.69 × 10^5^ n^3/2^ C_0_D^1/2^ ν^1/2^, (1)

where J_p_ is the peak current density in unit area (working area equals to 3.5 × 4 cm^2^), including the J_pa_ peak current density at oxidation and J_pc_ peak current density at reduction, n is the number of electrons (assumed to be 1), C_0_ (0.5 mol/L) is the concentration of the active ions in the electrolyte solution (in mol·cm^−3^), D is the diffusion coefficient of Li ions, and v is the potential scan rate (mV/s). We elucidated the electrochemical and energy storage properties of the IrO_2_/ITO/glass or NiO/ITO/glass by constructing threeelectrode cells, which contained a working electrode (IrO_2_ film on ITO/glass or NiO film on ITO/glass), a counter-electrode (Pt mesh), and a reference electrode (Ag/AgCl) in a 0.5 M LiClO_4_/perchlorate (LiClO_4_/PC) solution. The electrode comparison of CV curves on IrO_2_/ITO and NiO/ITO films with various Ar/O_2_ gas-flow ratios (1/2, 1/2.5, and 1/3) was proceeded at the 25th cycle based on a linear potential sweep ranging between −0.5 V and 2.0 V. The CV curves at the 25th cycle are shown in Figure 2 [33,34]. Furthermore, the diffusion of Li^+^ ions in the electrodes was determined by calculating the diffusion coefficients (D). The J_pa_, J_pc_, and the diffusion coefficient (D) values are summarized in Table 3. In Figure 2, CV curves demonstrated the enclosed area of IrO_2_ films were bigger than the NiO at all Ar/O_2_ gas ratios; moreover, the higher Ar/O_2_ gas ratios led to the larger enclosed area of the two material electrode films. Note that the device showed a significant capacitive behavior and indicated the participation of more ions in the electrochemical redox process. According to Table 3 and Figure 3, sample 3 (IrO_2_ with an Ar/O_2_ ratio of 1/3) presented the highest ion diffusion coefficients (D) of 1.09 × 10^−10^ cm^2^/s (oxidation)/1.10 × 10^−10^ cm^2^/s (reduction) in the IrO_2_ film, and sample 6 (NiO with Ar/O_2_ ratio is 1/3) presented the highest ion diffusion coefficients (D) of 1.93 × 10^−11^ cm^2^/s (oxidation)/3.12 × 10^−11^ cm^2^/s (reduction) in the NiO film. The higher diffusion coefficients represented a larger contact area and greater porosity, resulting in faster ion insertion/extraction, which was good for the transportation of Li ions.

### 3.2. Material Structure and Surface Morphology Analysis

**Figure 4:**
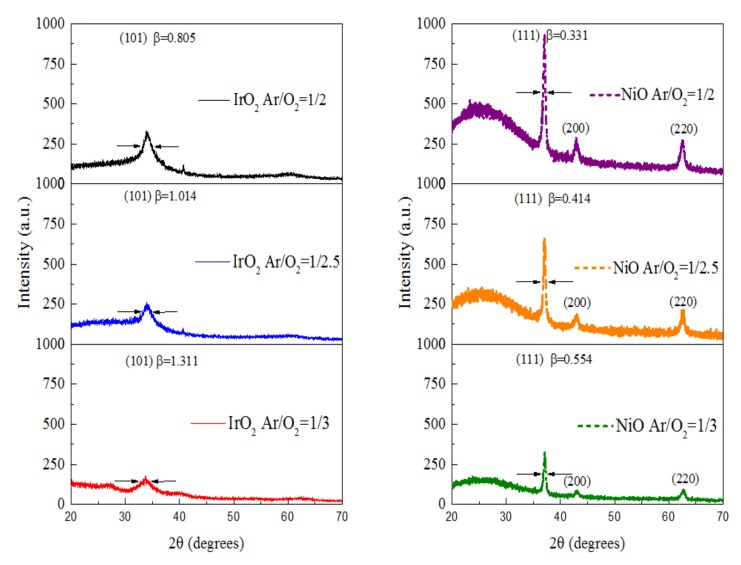
X-ray diffraction of IrO_2_ and NiO electrodes at various Ar/O_2_ mixing ratio.

To evaluate the crystal material structure and the possible phase change during the deposition process of both the IrO_2_ and the NiO electrode films with various Ar/O_2_ mixing ratios, the XRD diffraction patterns are shown in Figure 4; they were used to distinguish the crystalline nature and calculate the particle grain size. We acquired these structures and phase compositions by means of a comparison of the Joint Committee on Powder Diffraction Standard (JCDPS). After the subtraction of the diffraction peaks, the IrO_2_ electrode (JCPDS card no. 15-0870) main peak was located at a 2θ angle of 34° which could be indexed as the preferential plane of (101); the NiO electrode (JCPDS card no. 47-1049) peaks located at 2θ~37°, 43° and 63° were indexed as the preferential planes of (111), (200), and (220), respectively. The (111) preferential planes were different from the preferred (200) and (220) growth [36,37]. In Figure 4, at various angles for the two types of electrodes, we observed that the NiO electrode samples were more crystalline than the IrO_2_ electrode ones and that the intensity of diffraction decreased with an increase in the Ar/O_2_ mixing ratio.

However, the highly crystalline structure of the material is less favorable for the insertion/extraction of Li ions because to the compact atom structure. Moreover, the facets (101) and (111) had more noticeable crystallinity of the IrO_2_ and NiO electrodes. The average grain size (d) for (101) of the IrO_2_ electrode and (111) of the NiO samples was calculated using Scherrer’s equation [36]:

D = K λ/β cosθ(2)

where d is the average grain size, is the dimensionless shape factor, λ is the X-ray wavelength, β is the full width at half maximum (FWHM) of the X-ray diffraction peak in line broadening in radians, θ is the diffraction angle. The measured average grain sizes are presented in Table 4.

The average grain size decreased with an increase in the Ar/O_2_ mixing ratio of the two electrode samples. In contrast to the grain size, IrO_2_ electrode got the smaller size than NiO as the larger FWHM in Table 4. This study is to analyze surface morphology and optical and electrochromic properties f metal-oxide films based on various Ar/O_2_ gas-flow ratios [13,14,32]. In Figure 2, we found that with increase in oxygen flow rate the current density starts decreasing during CV analysis under same voltage applied condition. The reason for this may be attributed to decrease in the number of incident CAP gas ions on the NiO or IrO_2_ with increase in oxygen flow rate. Herein, we observed that IrO_2_ (sample #3) had smaller grain size of 6.35 nm at the Ar/O_2_ mixing ratio of 1/3 than NiO (sample #6) did (15.15 nm). The IrO_2_ electrode with the smallest grain size of 6.35 nm demonstrated the highest diffusion coefficient, as the Li ions transferred in a less hindered film than the NiO electrode. The smaller grain size was desirable in Table 4 as it offered more grain boundaries, which increased the diffusion coefficient. The CV diagram and the diffusion coefficient (D) were related to the grain size, and the behavior of the electrodes was in good agreement with that in the case of a relatively small grain size [36]. The decreasing grain size was associated with the increasing enclosed area of the corresponding CV. The enclosed area was as follows: Ar/O_2_ gas-flow ratios (1/2, 1/2.5, and 1/3): (1) IrO_2_ at 1/2 (7.14 mC/cm^2^), IrO_2_ at 1/2.5 (9.03 mC/cm^2^), IrO_2_ at 1/3 (11.05 mC/cm^2^), and (2) NiO at 1/2 (3.24 mC/cm^2^), NiO at 1/2.5 (5.13 mC/cm^2^), NiO at 1/3 (6.09 mC/cm^2^).

**Figure 5:**
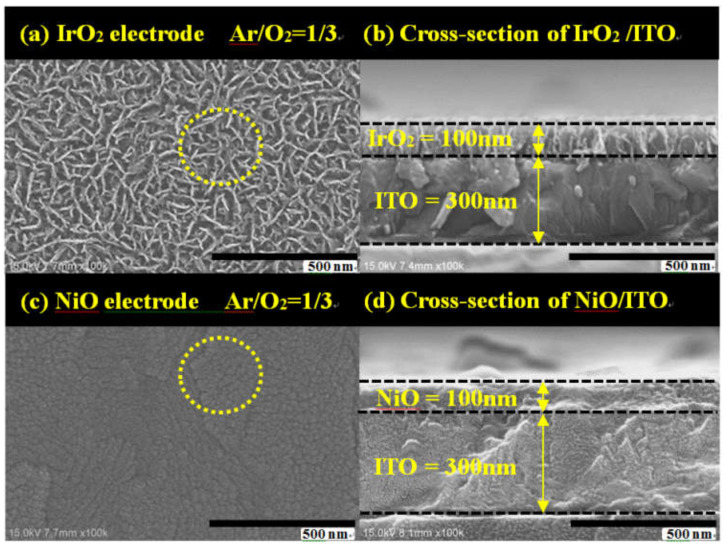
SEM images of surface morphology (**a**) IrO_2_ electrode with Ar/O_2_ = 1/3 (**c**) NiO electrode with Ar/O_2_ = 1/3; Cross-section morphology of (**b**) IrO_2_ electrode with thickness 100 nm (**d**) NiO electrode with a thickness of 100 nm.

**Figure 6:**
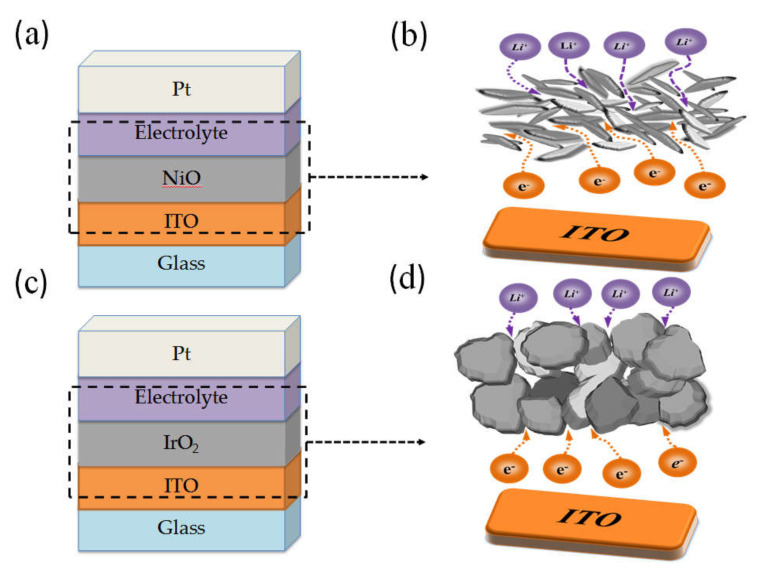
The schematics of Li ions path through surface morphology with different grain type (**a**) IrO_2_ electrode device (**b**) IrO_2_ electrode (**c**) NiO electrode device (**d**) NiO electrode.

Furthermore, Figure 5a,c show that correlate SEM surface morphology images IrO_2_ and NiO electrodes with 1/3 ratio of Ar/O_2_ at thickness 100 nm; Figure 5b,d show the cross-sectional SEM; the electrodes specimen was prepared on coated ITO 300 nm/glass. Therefore, the grain size and surface morphology should be regarded as essential factors in a study on the fabrication of ECDs. Our results of IrO_2_ films leaded to greatly diffusion coefficient could be attributed a porous structure that it revels surface morphology of grains which is like filamentary and interconnect. In Figure 6a,c show electrode device, Figure 6b,d are the grains skeleton indicated areas where adopted in Figure 6a,c dotted line. Tthe schematics explain the Li ion path through different grain types in the IrO_2_ and NiO surface morphology of an electrochromic film. This could be attributed to the grain types of filamentary and interconnected shape with a larger inner-pore structure on the IrO_2_ electrode, which rendered a larger contact area and greater porosity, resulting in the Li ions having sufficient time and space to insert into/extract from the interface. In addition, the grain types provided higher diffusion coefficients.

### 3.3. Optical Transmittance and Cycle Durability Analysis

**Figure 7:**
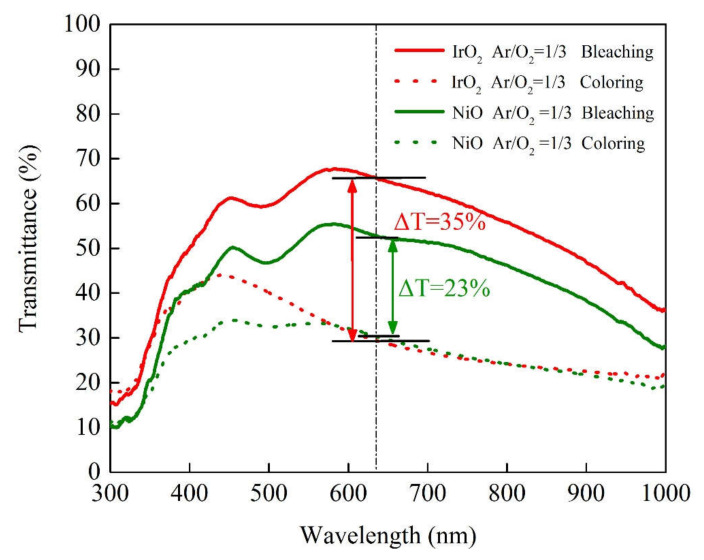
The optical transmittance spectra showing coloring and bleaching comparison states of the IrO_2_ and NiO electrodes with Ar/O_2_ = 1/3 in the range from 300 nm to 1000 nm.

**Figure 8:**
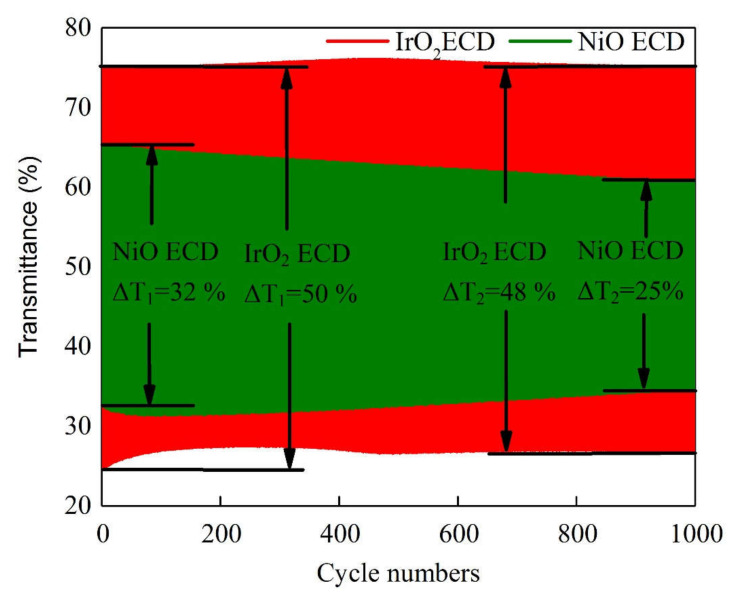
The comparison durability of the electrochromic device (ECD) is evaluated by optical transmittance modulation at 633 nm during 1000 cycles.

**Figure 9:**
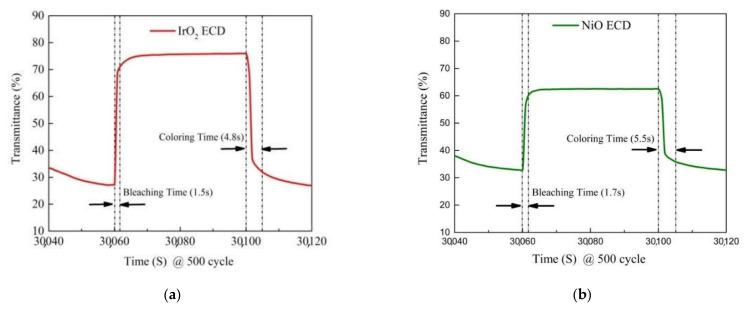
(**a**) IrO_2_ ECD and (**b**) NiO ECD: Switch response time for one single bleaching and coloring states @ 500 cycle (30040→30120 s).

The optical transmittance measurement of IrO_2_/ITO/glass and NiO/ITO/glass is helpful to understand the transmittance optical modulation (∆T = T_bleached_ − T_colored_) at a fixed wavelength of 633 nm, along with the switching response time and durability of the electrochromic devices (ECD). In Figure 7, we first elucidate the electrochemical properties of the IrO_2_/ITO/glass and NiO/ITO/glass by constructing three-electrode cells, which consisted of a working electrode (IrO_2_ film on ITO/glass and NiO film on ITO/glass), a counter-electrode (Pt mesh) and a reference electrode (Ag/AgCl) in a 0.5-M LiClO_4_/perchlorate (LiClO_4_/PC) solution. In Figure 7, the optical transmittance spectra of IrO_2_/ITO/glass and NiO/ITO/glass were carried with from 1.5 to −0.3 V versus AgCl/Ag with an Ar/O_2_ ratio of 1/3 at 633 nm. In our results, IrO_2_/ITO/glass had a higher optical transmittance modulation, ∆T = 35% (from T_bleaching_ (65%) to T_coloring_ (30%)), than the NiO electrode, ∆T = 23% (from T_bleaching_ (53%) to T_coloring_ (30%)). Note that the modulation of the optical transmittance of the 100-nm-thick IrO_2_ film with an Ar/O_2_ ratio of 1/3 was higher than that of the other samples, as indicated by the larger enveloped area in the CV curve in Figure 2 and Figure 7 show that the coloration and bleaching state of ECDs, as analyzed during a continuous potential from −2 V (coloration potential, Vc) to 2 V (bleaching potential, Vb), were measured by the CA curves and the in-situ optical response of transmittance at 633 nm. The coloration and bleaching of the switching times and speed were very important factors for the ECD system; it is defined as the time required for a 90% change in the full transmittance modulation [8]. Figure 8 presents the electrochromic performance of optical transmittance modulation at 633 nm after 1000 cycles. It demonstrated that IrO_2_-ECD (glass/ITO/WO_3_/liquid electrolyte/IrO_2_/ITO/glass) ∆T_1_ = 50% (from T_bleaching_ (75%) to T_coloring_ (25%)) was higher than that of NiO-ECD (ITO/NiO/liquid electrolyte/WO_3_/ITO) ∆T_2_ = 32%. In the 1000-cycle durability analysis, the optical transmittance indicated that IrO_2_-ECD demonstrated excellent durability, which remained as 96% of the original state value, as the IrO_2_ electrode had more transferred Li ions and outperformed NiO-ECD (78% of the original value). Figure 9a,b show the switching response time, including the bleaching and the coloration time in the middle of a long durability cycle test (@500 cycles). Figure 9a shows that in the case of IrO_2_-ECD, the switching response time was 1.5 s from the colored state to the bleached state and 4.8 s from the bleached state to the colored one. The IrO_2_-ECD in Figure 9a was faster than the NiO-ECD in Figure 9b, which took 1.7 s to switch from the coloration state to the bleaching state and 5.5 s to switch from the bleaching state to the coloration one. The faster switching speed was associated with the higher diffusion coefficients and the filamentary shape of the interface in the case of the IrO_2_ electrode, which facilitated the transfer of the Li ions into/out of interface electrodes and the electrolyte. The IrO_2_-ECD showed higher optical transmittance modulation than NiO-ECD which was useful for promoting electrochromic application for cycle durability test. A comparison of the electrochromic and optical properties obtained in this study which was reported in previous research and presented in Table 5.

## 4. Conclusions

In conclusion, we presented a comparison of electrodes on IrO_2_/ITO and NiO/ITO films with various Ar/O_2_ gas-flow ratios in ECDs and investigated the electrochemical, structural, and optical properties. We developed electrochromic electrodes by using the CAP technique as an alternative method to fabricate ECDs with a high deposition rate and at a low cost. We observed that the IrO_2_ electrode films with a filamentary surface morphology and an Ar/O_2_ ratio of 1/3 (sample 3) demonstrated the highest ion diffusion coefficients (D) of 1.09 × 10^−10^ cm^2^/s (oxidation)/1.10 × 10^−10^ cm^2^/s (reduction) and the smallest grain size of 6.35 nm. The electrochromic performance of IrO_2_-ECD (glass/ITO/WO_3_/liquid electrolyte/IrO_2_/ITO/glass) for the optical transmittance modulation, ∆T = 50% (from T_bleaching_ (75%) to T_coloring_ (25%)) at 633 nm, was higher than that of NiO-ECD, ∆T = 32%, after 1000 cycles. The IrO_2_-ECD demonstrated excellent durability after 1000 cycles, which remained at 96% of the original value, and outperformed NiO-ECD (78% of the original value). We found that IrO_2_-ECD had a switching response time of 1.5 s from the coloration state to the bleaching state and 4.8 s from the bleaching state to the coloration one. The fast response time of the IrO_2_ electrode facilitated the transfer of Li ions into/out of the interface electrodes and the electrolyte, owing to the higher diffusion coefficients and the filamentary shape of the interface. Therefore, we concluded that IrO_2_ -ECD is promising for electrochromic applications to a cycle durability test, as an alternative to NiO-ECD.

## Figures and Tables

**Table 1:** Deposition parameters of IrO_2_ and NiO electrode films.

No.	Electrode	Ar/O_2_ (Ar = 20 sccm)	W.P. (Torr)	DC Power (W)	Deposition Temp (°C)	Deposition Time (s)	Thickness (nm)
Sample 1	IrO_2_	1/2	1.2 × 10^−3^	1250	100	40	100
Sample 2	IrO_2_	1/2.5	1.7 × 10^−3^	1250	100	40	100
Sample 3	IrO_2_	1/3	1.9 × 10^−3^	1250	100	40	100
Sample 4	NiO	1/2	1.2 × 10^−3^	1250	100	100	100
Sample 5	NiO	1/2.5	1.7 × 10^−3^	1250	100	100	100
Sample 6	NiO	1/3	1.9 × 10^−3^	1250	100	100	100

**Table 2:** Deposition parameters of transparent ITO glass and WO_3_ electrode film.

Target	W.P. (Torr)	Ar/O_2_ (sccm)	DC power (W)	Deposition Time (min)	Deposition Rate (nm/min)	Deposition Temp °C	Thickness (nm)
ITO	3 × 10^−3^	1/3 (Ar = 100)	500	60	5	200	300
W Metal	8 × 10^−3^	1/3 (Ar = 100)	1500	15	13.3	50	200

**Table 3:** Diffusion coefficients of IrO_2_ and NiO electrodes with various Ar/O_2_ mixing ratios.

No.	Electrode	Ar/O_2_ (Ar = 20 sccm)	Anodic Current (j_p__a_)	Cathodic Current (j_pc_)	D for j_pa_ Oxidation	D for j_pc_ Reduction
Sample 1	IrO_2_	1/2	2.82 × 10^−4^	2.83 × 10^−4^	4.40 × 10^−11^	4.42 × 10^−11^
Sample 2	IrO_2_	1/2.5	3.53 × 10^−4^	3.45 × 10^−4^	6.88 × 10^−11^	6.57 × 10^−11^
Sample 3	IrO_2_	1/3	4.44 × 10^−4^	4.47 × 10^−4^	1.09 × 10^−10^	1.10 × 10^−10^
Sample 4	NiO	1/2	8.70 × 10^−5^	1.49 × 10^−4^	4.18 × 10^−12^	1.22 × 10^−11^
Sample 5	NiO	1/2.5	1.65 × 10^−4^	2.16 × 10^−4^	1.50 × 10^−11^	2.59 × 10^−11^
Sample 6	NiO	1/3	1.87 × 10^−4^	2.38 × 10^−4^	1.93 × 10^−11^	3.12 × 10^−11^

**Table 4:** Average grain size of IrO_2_ and NiO electrode.

No.	Electrode	Ar/O_2_ (sccm)	2θ (deg)	FWHM (β)	Ave Grain Size (nm)
Sample 1	IrO_2_	1/2	34.832°	0.805°	10.03
Sample 2	IrO_2_	1/2.5	34.644°	1.014°	8.20
Sample 3	IrO_2_	1/3	34.312°	1.311°	6.35
Sample 4	NiO	1/2	37.815°	0.331°	25.30
Sample 5	NiO	1/2.5	37.613°	0.414°	20.27
Sample 6	NiO	1/3	37.282°	0.554°	15.15

**Table 5:** Comparison of our results with the literature on various materials and methods [8,10,13,37,38,39].

Materials/Device	Method	∆T (%)	CE (cm^2^/C)	Switching Time (t_c_/t_b_)	Ref.
WO_3_/IrO_2_	CAP	50	-	4.8/1.5 s	This work
WO_3_/NiO	CAP	46	90	3.1/4.6 s	[8]
WO_3_/NiO	DC Sputtering	55	87	10/20 s	[37]
WO_3_/PANI	Electro polymerization	37.4	98.4	9.9/13.6 s	[38]
WO_3_/PANI	Dip-coating	54.3	79.7	1.4/1.1 s	[10]
WO_3_	Spray	-	-	-	[13]
(NH_4_)_0_._33_ WO_3_	Hydrothermal	60.9	60.9	5.7/4.2 s	[39]

## Data Availability

The data presented in this study are available on request from the corresponding author. The data are not publicly available due to privacy.
